# Factor validation of the International Trauma Questionnaire in a sample of trauma-exposed Colombian adults in the MI-VIDA study – RETRACTION

**DOI:** 10.1192/bjo.2025.10928

**Published:** 2025-12-01

**Authors:** Martin Robinson, Donncha Hanna, Natasha Bloch, Chérie Armour

The Editors of BJPsych Open and the authors have retracted the following article:

Factor validation of the International Trauma Questionnaire in a sample of trauma-exposed Colombian adults in the MI-VIDA study.

In July 2025, the authors of this paper voluntarily acknowledged a coding error in the analysis file that impacted the descriptive statistics presented in the *ICD-11 Diagnostics* subsection of the Results leading to incorrect rates of PTSD and CPTSD probable diagnosis reported in Table 3. This error meant that the transformation for symptom endorsement (response ≥2) was not correctly applied to each item, hence the calculation of symptom endorsement and probable diagnosis rates was mistakenly applied to raw scores.

The Editors reviewed the changes and noted that the numbers detailed in Table 3 had changed significantly and there had been resulting changes in the *Discussion* and *Strength and limitations* sections and a significant impact on the results.

In view of the extent and significance of the errors, the Editors concluded that a corrigendum would not be sufficient to resolve these issues. They agreed that retraction of the original article with subsequent resubmission of a corrected paper would be the best course of action.

The journal did not request an institutional investigation.

All authors have agreed to this retraction.
